# *Staphylococcus aureus* serine protease-like protein B elicits a type 1/type 2 immune response in atopic dermatitis patients

**DOI:** 10.3389/fimmu.2026.1798583

**Published:** 2026-06-04

**Authors:** Rebecca Pospich, Goran Abdurrahman, Tatjana Honstein, Maria Nordengrün, Stephan Traidl, Gabriele Begemann, Petra Kienlin, Thomas Werfel, Barbara M. Bröker, Lennart M. Roesner

**Affiliations:** 1 Department of Dermatology and Allergy, Hannover Medical School, Hannover, Germany; 2 Institute of Immunology, University Medicine Greifswald, Greifswald, Germany; 3 Cluster of Excellence RESIST (EXC 2155), Hannover Medical School, Hannover, Germany

**Keywords:** adaptive immune system, antigen, atopic dermatitis, inflammation, phenotype, skin, *Staphylococcus*, T cell

## Abstract

**Introduction:**

Atopic dermatitis (AD), a common chronic inflammatory skin disease, is characterized by type-2-mediated inflammation, along with the detection of type-1 and type-3 cytokines in lesional skin. The skin microbiome of lesional skin is dominated by the pathogen *Staphylococcus aureus*, which can aggravate the disease via pathogenicity factors. To elucidate the impact of the adaptive immune response on inflammation in AD, this study focused on staphylococcal serine-like proteases (Spl) of *S. aureus*, a family of secreted pathogenicity factors with the potential to induce type-2 responses.

**Methods:**

Specific serum IgE against Spl family members was quantified, and SplB-specific CD4^+^ T cells were identified by surface expression of CD154 after *in vitro* stimulation with recombinant SplB. Immunodominant epitopes within the SplB primary structure were predicted to generate MHC multimers for staining, sorting, and cytokine analysis of SplB-specific T cells. TCRB sequencing was applied to identify SplB-specific T cells in AD skin lesions.

**Results:**

We observed significantly elevated levels of IgE antibodies specific for Spl family proteins in patients with AD compared to healthy controls. *In vitro*, recombinant SplB was sufficient to induce T cell activation and cytokine secretion in PBMCs from patients with AD and healthy controls. SplB-specific T helper cells, which were cell-sorted from patients’ blood by MHC-II multimers, showed the capacity to produce IFN-γ and IL-13 *ex vivo*. Clonal propagation of specific T cells was confirmed by TCR sequencing, and SplB-specific TCR sequences were re-identified in autologous lesional skin biopsy material.

**Discussion:**

The presence of clonally propagated SplB-specific T cells in the skin of patients with AD strongly suggests an impact on inflammation. This type of cellular immune response, which is not exclusively polarized towards type 2, reflects the AD phenotype. This suggests that the adaptive immune response to S. aureus contributes to this phenotype.

## 1 Introduction

Atopic dermatitis (AD) is a common inflammatory skin disease affecting 10%–20% of children and approximately 1%–3% of adults (1, 2). Several factors contribute to the development of this disease, including epithelial barrier defects and Th2-skewed immune responses, leading to inflammation and pruritus (3). The major contributing cells are skin-infiltrating T cells, which produce Th2 and Th22 cytokines in the acute (4, 5) and Th1 cytokines in the chronic phase (6–8), whereas Th17 appears to play a role in certain endotypes (9, 10).

Skin colonization by the pathogenic bacterium *Staphylococcus aureus* (*S*. *aureus*), which is prevalent in patients with AD, especially at lesional skin sites, is associated with disease severity (11, 12). *S. aureus* directly contributes to disease pathology by producing virulence factors, modulating the immune response, and promoting skin barrier defects (13, 14). Staphylococcal enterotoxins (SE) act as superantigens and can directly activate T cells, dysregulate the immune response, and thereby drive the disease. α-toxin (α-hemolysin) can induce cytokine secretion by host cells when present at sublytic concentrations. These and other secreted virulence factors can reach the deeper layers of the skin, resulting in tissue destruction and T cell activation (15–17). In addition to recognition by the innate immune system, *S. aureus* proteins, such as fibronectin-binding protein 1 and clumping factor A, can also act as classical T cell antigens (18, 19). Among these are extracellular proteases, such as serine proteases (V8 protease, exfoliative toxins A and B), a metalloprotease (aureolysin), and cysteine proteases (Staphopains A and B) (20). Proteases are often potent allergens, such as those found in pollen, house dust mites, and fungi (21). Humoral adaptive immune responses have been observed to be biased towards IgE in patients with allergies (22), and *S. aureus* is considered a possible promoter of type-2-biased immune reactions (23, 24).

In this study, we focused on *S. aureus*-derived Spls, which are a group of six serine proteases (SplA-SplF) encoded by the *spl* operon (25, 26), which were among the top hits in a systematic approach to identify IgE-reactive proteins capable of inducing allergic reactions within the *S. aureus* secretome. In that study, Spl-specific IgE antibodies were found in most patients with asthma, but only in a minority of healthy participants (27), making it a model antigen family to investigate a potential factor in the exacerbation of type-2 inflammatory diseases, such as AD.

The *splb* gene was found in 54.2% of *S. aureus* isolates from patients with wound infections (13 of 24), in 46.7% of isolates from skin infections (seven of 15), and in 77.7% of healthy individuals (seven of nine) (28). Among all *S. aureus* isolates tested, 84% contained at least one Spl-encoding gene, suggesting that the proteases confer an evolutionary advantage. Spls have been shown to promote a type 2 biased immune response in healthy adults, patients with asthma, and those with cystic fibrosis, and contribute to pneumonia and allergic asthma (27, 29–31).

SplB has also been reported to modulate host immune functions by cleaving complement proteins and deubiquitinating enzymes (32, 33). These findings suggest that SplB may shape host immune responses during *S. aureus* colonization.

In this study, we tested the hypothesis that the adaptive immune response to Spls is type 2 driven and thereby influences inflammation in AD. We measured specific IgE and identified T-cell peptide epitopes in SplB and used this knowledge for MHC multimer staining to study the prevalence of SplB-specific T cells in lesional AD skin and characterize them by dual TCRVB sequencing. While we observed a specific T-cell response expressing type 1 and type 2 cytokines in both patients and controls, AD patients showed an increased prevalence of specific IgE in the serum. Finally, we detected clonally propagated SplB-specific T cells in lesional skin, suggesting a direct contribution to skin inflammation and the chronification of the disease.

## 2 Methods

### 2.1 Participant enrollment

Blood samples were collected from adult patients with AD and healthy individuals. Patients were recruited from the Department of Dermatology and Allergy of Hannover Medical School (MHH) and diagnosed with mild-to-severe AD according to the criteria of Hanifin and Rajka (34). Patients who had used systemic corticosteroids, immunosuppressants, phototherapy, or allergen-specific immunotherapy within three weeks prior to the study were excluded. Further exclusion criteria were pregnancy and breastfeeding. Disease severity was assessed using the SCORing Atopic Dermatitis (SCORAD) severity score. IgE and SCORAD data are listed in **Supplementary Table 1**. From the five recruited patients, 4 mm punch biopsies were taken from lesional inflamed skin (ages 29–53, mean age, 37.8, 60% female, 40% male). This study was performed in accordance with the Declaration of Helsinki and approved by the Ethics Committee of Hannover Medical School (No. 7565). All participants recruited gave their written informed consent prior to the study.

### 2.2 Characterization of HLA-genotype

Isolated DNA of from PBMCs of patients with AD was used to characterize the HLA type using next generation sequencing, as described elsewhere (35). Briefly, next-generation sequencing was performed using an Illumina Sequencing panel covering the genes for HLA-A, HLA-B, HLA-C, HLA-DPB1, HLA-DQA1, HLA-DQB1, and HLA-DRB1.

### 2.3 Recombinant SplB protein and SplB peptides

Recombinant *S. aureus* SplB was produced in *Bacillus subtilis* strain 6051HGW LS8P-D, as described previously (27). Briefly, protein expression was induced *in vitro*, and SplB was purified using ion-exchange chromatography with an SP Sepharose Fast Flow column (GE Healthcare). The Spl-containing fractions were identified by SDS-PAGE and concentrated using centrifugal filter units (Amicon Ultra 30K/10K, Merck Millipore). The quality of the resulting SplB was verified using SDS-PAGE. SplB peptides were synthesized by Peptides and Elephants with a purity of >90% (Hennigsdorf, Germany).

### 2.4 IgE ELISA

To measuring human serum IgE levels, ELISAs were performed as previously described (27). Briefly, 96-well plates were coated with 100 µg of antigen solution (5 µg/mL) overnight at 4 °C. The plates were washed three times with Dulbecco’s phosphate-buffered saline w/o Ca, Mg (PBS; PAN-Biotech GmbH, Aidenbach, Germany) containing 0.05% Tween, and free binding sites were saturated by incubating the wells with 150 μL of blocking buffer (10% fetal calf serum [FCS; Life Technolologies, Karlsruhe, Germany] in PBS). After blocking, the wells were incubated with 50 μL of 1:5 human serum diluted in blocking buffer. To measure IgE levels, a biotin-conjugated secondary antibody (mouse anti-human IgE, 10 μg/mL; Invitrogen; clone HP6029) was used in combination with peroxidase-conjugated streptavidin (3 μg/mL; Dianova, Hamburg, Germany) to detect antibody binding. Single OD measurements were performed at 450 nm, and the blank value (in the absence of serum) was multiplied by 1.5 and subtracted.

### 2.5 CD154 assay

PBMCs (1 × 10^6^) were stimulated with recombinant SplB (2.5 µg/mL), SEB (1 µg/mL, S4881-1MG, Sigma-Aldrich, St. Louis, MO, USA), or tetanus toxoid (1:1,000, Mérieux, Sanofi Pasteur MSD, Lyon, France). To prevent CD154 internalization and provide co-stimulation and a survival signal, agonistic antibodies against CD40 (1 µg/mL, 130-094-133, clone HB14, Miltenyi, Bergisch Gladbach, Germany) and CD28 (0.5 µg/mL, 555726, clone CD28.2, Becton Dickinson GmbH, Franklin Lakes, NJ, USA) were added for 24 h. To investigate SplB-specific T cells, PBMCs were stained with antibodies (unless otherwise stated: from BioLegend, San Diego, CA, USA): PerCP-conjugated anti-human CD4 (317432, clone OKT4) or PE-Cy5-conjugated anti-human CD4 (555348, clone RPA-T4, Becton Dickinson GmbH, Franklin Lakes, NJ, USA) BV510-conjugated anti-human CD14 (301842, clone M5E2), BV510-conjugated anti-human CD19 (302242; clone HIB19), FITC-conjugated anti-human CD154 (310804; clone 24-31), APC-conjugated anti-human CXCR3 (FAB160A, clone 49801, R&D Systems Inc, Minneapolis, MN, USA), BV421-conjugated anti-human CCR4 (359414, clone L291H4), PE-Cy7-conjugated anti-human CCR6 (353418, clone G034E3), and PE-conjugated anti-human CCR10 (130-120-407, clone: REA326, Miltenyi, Bergisch Gladbach, Germany) (36). For live/dead discrimination, cells were stained with a fixable viability dye (FvD-506, 65-0866-14, Invitrogen™, Thermo Fisher, Waltham, MA, USA). Only cells from donors that showed higher frequencies of viable CD4^+^CD154^+^ lymphocytes were subjected to subsequent surface marker analysis for cellular characterization. Flow cytometry data were collected using a BD FACSCanto™ II flow cytometer with BD FACSDiva™ software (version 8.0.1) and analyzed with Kaluza software (Beckman Coulter).

### 2.6 CD137 assay

PBMCs (1 × 10^6^) were stimulated with SplB (2.5 µg/mL), SEB (1 µg/mL, S4881-1MG, Sigma-Aldrich, St. Louis, MO, USA), or tetanus toxoid (1:1,000, Mérieux, Sanofi Pasteur MSD, Lyon, France) for 24 h. To investigate SplB-specific CD8^+^ T cells, PBMCs were stained with antibodies (unless stated otherwise: from BioLegend, San Diego, CA, USA): BV510-conjugated anti-human CD14 (301842, clone M5E2), BV510-conjugated anti-human CD19 (302242, clone HIB19), FITC-conjugated anti-human CD8 (301050, clone RPA-T8), PerCP/Cy5.5-conjugated anti-human CD137 (309814, clone 4B4-1). For live/dead discrimination, cells were stained with a fixable viability dye (FvD-506, 65-0866-14, Invitrogen™, Thermo Fisher, Waltham, MA, USA). Flow cytometry data were collected using a BD FACSCanto™ II flow cytometer with BD FACSDiva™ software (version 8.0.1) and analyzed with Kaluza software (Beckman Coulter).

## 3 Generation of T cell lines

PBMCs (1 × 10^6^) were either stimulated with 2.5 µg/mL SplB or left not stimulated (n.s.) and cultivated for three weeks. On day 7, rhIL-2 (10 U/mL; Merck, Darmstadt, Germany) was added. On day 14, cells were expanded with 1 × 10^6^ allogeneic, irradiated (55 Gy) PBMCs as feeder cells in the presence of phytohemagglutinin (10 µg/mL, PHA, Merck, Darmstadt, Germany).

### 3.1 T-cell receptor Vβ sequencing and analysis

Sequencing of the complementarity-determining region 3 (CDR3) of the T cell receptor beta (TCRB) gene was performed using the ImmunoSEQ™ human T cell receptor beta (hsTCRB) kit according to the manufacturer’s instruction (Adaptive Biotechnologies^®^, Seattle, WA, USA). The assay combines a multiplex PCR using primers that bind to the V and J gene segments of target genomic DNA, followed by a second PCR with a unique barcode primer and high-throughput sequencing using Illumina MiSeq and NextSeq analyzers (Illumina). Sequencing was performed according to the manufacturer’s instruction with 100,000 reads (survey resolution), including V, D, and J segments, and nonproductive sequences were filtered out. TCRB CDR3 data were analyzed using the ImmunoSEQ Analyzer 3.0 (Adaptive Biotechnologies, Seattle, WA, USA) for data cleaning and to detect overlapping CDR3 sequences between lesional AD skin and SplB-specific T cell lines.

### 3.2 *In silico* prediction of potential immunodominant SplB epitopes

9-mer and 15-mer peptides were selected by applying two T cell epitope prediction algorithms, SYFPEITHI (37) and the consensus algorithm of the Immune Epitope Database (IEDB) (38), to the SplB sequence from *S. aureus*. MHC binding to HLA-A*02:01 (9-mer peptides) and HLA-DRB1*01:01, DRB1*04:01, DRB1*11:01, DRB1*13:01, and DRB1*15:01 (15-mer peptides) was taken into account, as these allelles occur at relatively high frequencies in the German population (39, 40).

### 3.3 SplB 9-mer peptide HLA-A*02:01 complex formation and tetramer construction

The binding characteristics of predicted 9-mer peptides to HLA-A*02:01 were investigated using the immunAware kit, following the manufacturer’s instructions (immunAware, Copenhagen, Denmark). CMVpp65_495–503_ was used as a positive control.

### 3.4 HLA class I and II tetramer staining

Fresh PBMCs were isolated from AD patients with HLA-DRB1*15:01 and/or HLA-DRB1*11:01 and stimulated in the presence or absence of SplB (2.5 µg/mL) or SplB peptides (10 µg/mL). On day 7, rhIL-2 (10 U/mL) was added to the culture. On day 14, cells were harvested and stained with tetramers (15 nM–30 nM in IAB medium) at 37 °C for 1 h according to the manufacturer’s instruction (immunAware, Copenhagen, Denmark). Afterward, cells were stained with antibodies at 4 °C (BioLegend, San Diego, CA, USA): APC-conjugated anti-human CD4 (300537, clone RPA-T4), BV421-conjugated anti-human CCR4 (359414, clone L291H4), and PE-Cy7-conjugated anti-human CCR6 (353418, clone G034E3). For live/dead discrimination, cells were stained with a fixable viability dye (FvD-780, 65-0865-14, Invitrogen™, Thermo Fisher, Waltham, MA, USA). CD8^+^ T cells stained with HLA-A*02:01 tetramers were further stained with FITC-conjugated anti-human CD8 (3010056, clone RPA-T8) and for live dead discrimination, cells were stained with a fixable viability dye (FvD-506, 65-0866-14, Invitrogen™, Thermo Fisher, Waltham, MA, USA).

### 3.5 SplB structure prediction and alignment of SplA–SplF

For SplB protein structure prediction and to highlight the positions of the peptides SplB_51–65_ and SplB_99–113_ within the SplB protein, AlphaFold DB version 2022-11–01 was used (AlphaFold Data Copyright (2022) DeepMind Technologies Limited) (41, 42). Alignment of SplA–SplF was performed using CLC Sequence Viewer 6.6.2 (Qiagen, Venlo, Netherlands).

### 3.6 Statistics

For statistical analysis, GraphPad Prism^®^ 8.4.3 (GraphPad Software Inc., San Diego, CA, USA) was used. Data were analyzed for normal distribution using the Shapiro–Wilk-test. Unless stated otherwise, non-normally distributed data were analyzed using a two-tailed Mann–Whitney test for unpaired data and the Wilcoxon matched-pairs signed-rank test for paired data for single comparisons; for comparisons involving two or more groups, a Friedman test with Dunn’s multiple comparison posttest was used for paired data, and a Kruskal–Wallis test with Dunn’s multiple-comparison test was used for unpaired data.

## 4 Results

### 4.1 AD patients are sensitized to *S. aureus* serine protease-like proteins

**Figure 1:**
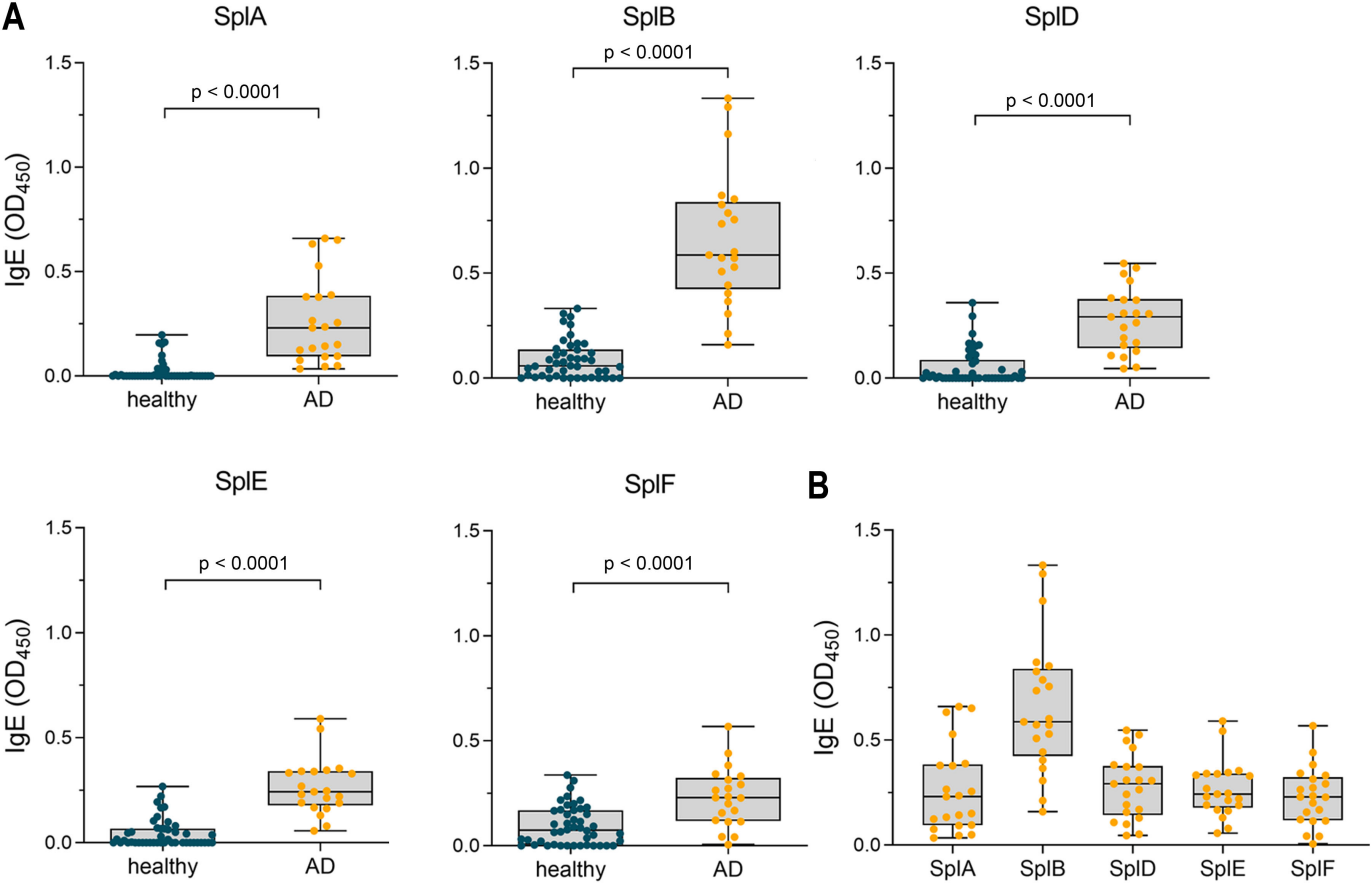
Increased serum IgE levels against Spls in AD. **(A)** IgE in the serum of AD patients (n = 21) and healthy individuals (n = 40) binding to SplA, SplB, SplD, SplE, and SplF were determined with ELISA. AD patients show increased IgE against all Spls. P-values are based on Mann–Whitney U test. **(B)** IgE against all Spls in AD patients showing increased IgE against SplB. P-values are based on Friedman test with Dunn’s multiple comparison test. Each data point reflects a single patient. Median with interquartile range are depicted.

Since the majority of allergic asthma patients have been shown to be IgE-sensitized to Spls, and AD patients are often colonized with *S. aureus*, we measured Spl-binding serum IgE in AD patients and healthy non-AD individuals. Spl-specific IgE levels were increased in patients with AD compared to healthy individuals for five Spls tested (SplA, SplB, SplD, SplE, SplF) (**Figure 1A**). When comparing IgE levels against the different Spls, SplB appeared to be immunodominant in AD patients (**Figure 1B**).

### 4.2 AD patients and healthy individuals harbor SplB-specific CD4^+^ T cells in the circulation, which predominantly express CCR4 and CCR6 on the cell surface

**Figure 2:**
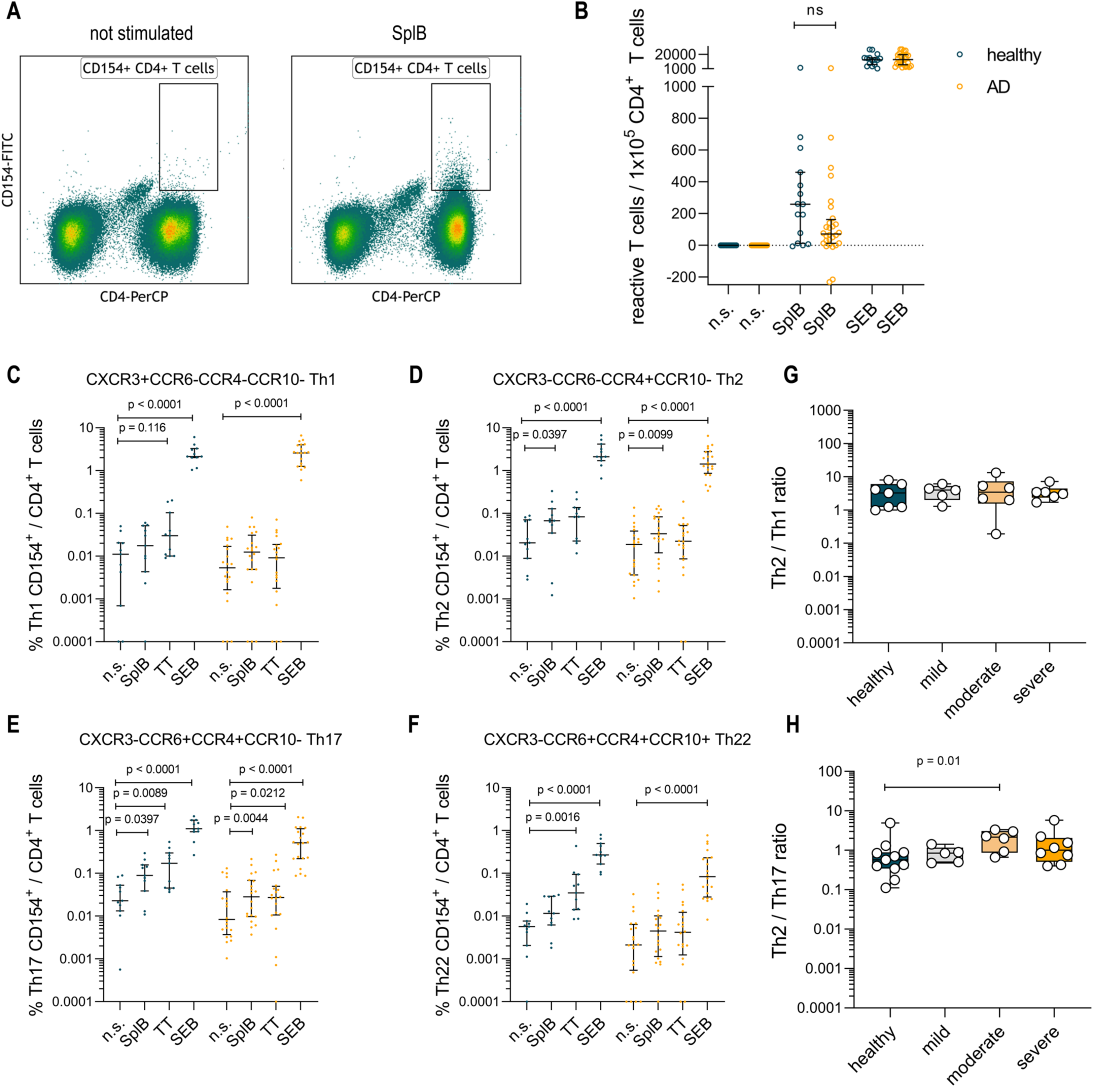
CD4^+^ T cells in the circulation react to SplB. PBMCs were isolated from healthy donors and AD patients. PBMCs (1 × 10^6^) were stimulated with SplB in the presence of anti-human CD40 and anti-human CD28 for 24 h. **(A)** Expression of CD154 on CD4^+^ T cells with or without stimulation with SplB gated on live CD4^+^ T cells. **(B)** SplB-reactive, CD154^+^ T cells per 1 × 10^5^ CD4^+^ T cells in healthy adults (n = 13, blue) and AD patients (n = 27, yellow); CD154^+^ T cell frequencies in unstimulated control cells were subtracted. P-values are based on Mann–Whitney U test. Median with interquartile range are depicted. (**C–F**) CD154^+^ CD4^+^ T cells were characterized by surface expression of CCR4, CCR10, CCR6, and CXCR3 to define **(C)** CD154^+^ Th1 (CXCR3^+^CCR4^−^CCR10^−^CCR6^−^), **(D)** CD154^+^ Th2 (CXCR3^−^CCR4^+^CCR10^−^CCR6^−^), **(E)** CD154^+^ Th17 (CXCR3^−^CCR4^+^CCR10^−^CCR6^+^), and **(F)** CD154^+^ Th22 (CXCR3^−^CCR4^+^CCR10^+^CCR6^+^) cells. Frequencies within 1 × 10^5^ CD4 T cells are shown. Friedman test with Dunn’s multiple comparison test. **(G)** Th2/Th1 and **(H)** Th2/Th17 ratio of SplB reactive CD4^+^ T cells in healthy individuals and AD patients with mild (SCORAD <25), moderate (SCORAD 25–50) and severe disease (SCORAD >50). Each data point reflects a single patient. Kruskal–Wallis test with Dunn’s multiple comparison test. TT, tetanus toxoid, SEB, *S. aureus* enterotoxin **(B)** Median with interquartile range are depicted.

To investigate the frequencies of SplB-specific T cells in the circulation of AD patients and healthy individuals, we assessed the upregulation of CD154 on CD4^+^ T cells after stimulation of PBMCs with recombinant SplB (**Figure 2A**). CD154, also known as CD40 ligand, is rapidly upregulated on CD4^+^ T cells after antigen recognition by the TCR (43). After stimulation with SplB, the frequency of CD154^+^CD4^+^ T cells increased in almost all subjects tested, and SplB-specific T helper cells were detected at frequencies of approximately 0.1% (median 0.07% in patients, 0.26% in controls) among CD4^+^ T cells after background correction. The frequency did not differ between patients and healthy controls and showed high interindividual variability, as expected (**Figure 2B**). Tetanus toxoid was used as a control to compare responses to a common recall antigen. The frequency of SplB-induced CD154^+^ T helper cells was in the range of tetanus toxoid–reactive T cells and was distinct compared to background levels (**Supplementary Figure 1**).

The expression of different chemokine receptors on the surface of CD4^+^ T cells is commonly used as a surrogate marker of CD4^+^ T cell subsets, including Th1, Th2, Th17, and Th22 cells (36). CD154^+^ cells were therefore subsequently characterized for the expression of cell surface CXCR3, CCR4, CCR6, and CCR10 (gating strategy, **Supplementary Figure 2**). We detected predominantly the marker combination described for Th2 cells (**Figure 2D**, CXCR3^−^CCR6^−^CCR10^−^CCR4^+^) and Th17 cells (**Figure 2E**, CXCR3^−^CCR6^+^CCR4^+^CCR10^−^) on SplB-specific T cells in both healthy individuals and AD patients. T cells displaying surface marker combinations commonly used as surrogates for Th1 (**Figure 2C**) or Th22 (**Figure 2F**) were not significantly increased after SplB stimulation. The Th2 phenotype was observed to be more pronounced than the Th17 phenotype, especially in moderately affected AD patients, which could represent a unique endotype with preferentially greater Spl reactivity (**Figure 2H**). The Th2/Th1 ratio was not altered in this set of measurements (**Figure 2G**).

### 4.3 Th1 and Th2 cytokines dominate the response to SplB in AD patients

**Figure 3:**
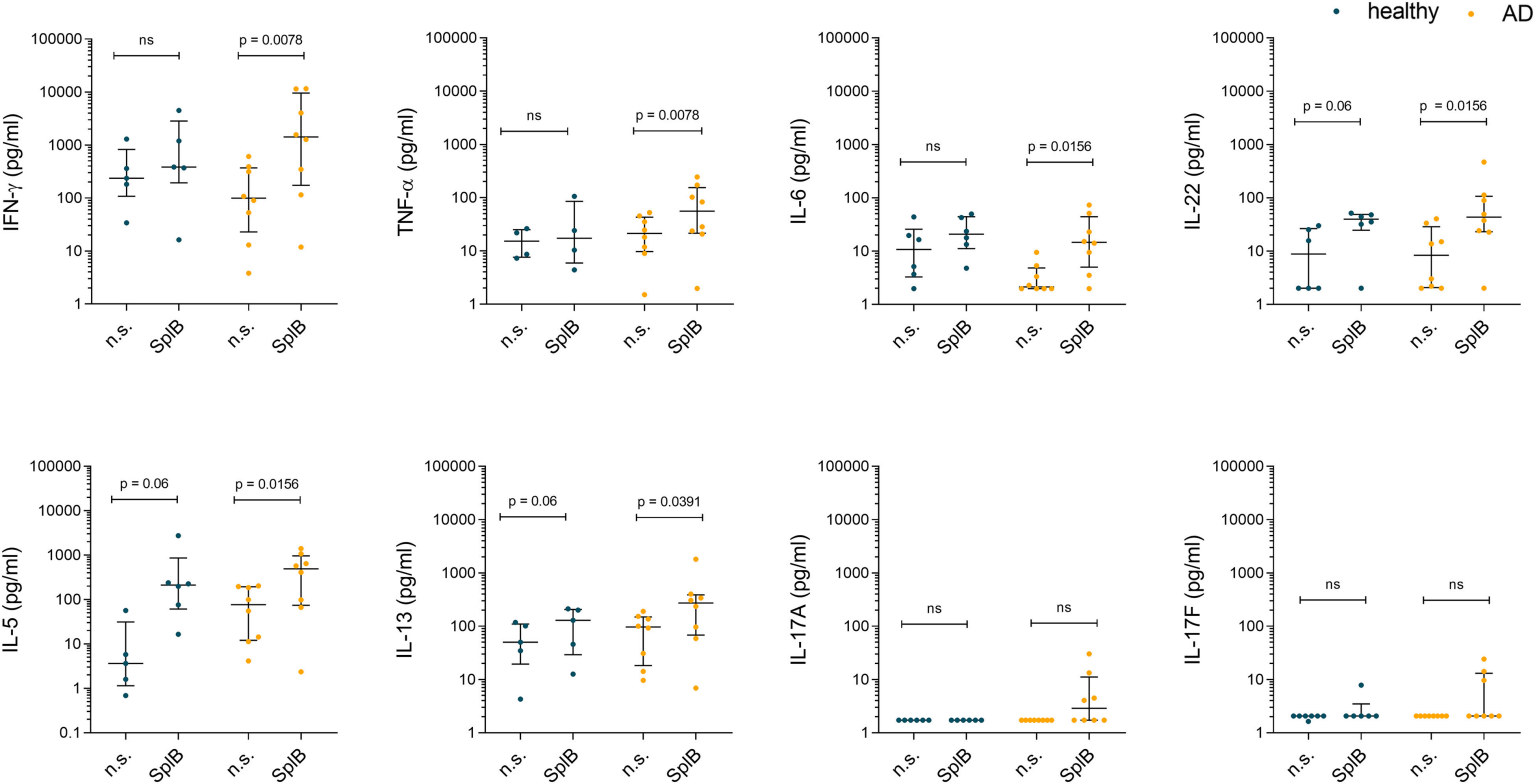
Cytokine secretion of SplB stimulated immune cells. T cells and monocytes were isolated from PBMCs of AD patients (n = 8, yellow) and healthy individuals (n ≥4, blue) and stimulated with recombinant SplB. Cytokine concentrations as depicted were measured after 9 days by bead-based cytometry assay. Wilcoxon test. Each data point reflects a single patient. Median with interquartile range are depicted.

To characterize the cytokine response to SplB in AD patients and healthy individuals, we isolated T cells and autologous antigen-presenting cells and stimulated them with SplB. AD patients and, to a lesser extent, healthy controls, showed a cytokine response that was dominated by the Th1 cytokines IFN-γ and TNF-α, the Th2 cytokines IL-5 and IL-13, as well as IL-22 (**Figure 3**). In contrast, the Th17 cytokines IL-17A and IL-17F did not reach meaningful levels, although the presence of IL-17A-secreting T cells could be confirmed by ELISpot (**Supplementary Figure 3**).

### 4.4 Identification of HLA-DRB1*11:01 and HLA-DRB1*15:01 restricted SplB epitopes in patients with AD

To exclude cell culture artifacts and characterize SplB-specific T cells without prior stimulation, we used bioinformatic epitope prediction and proliferation testing to construct SplB-peptide–MHC tetramers. Using the database SYFPEITHI and the epitope prediction algorithm IEDB (37, 38), we identified putative SplB epitopes for the five most common MHC class II HLA-DR types in the European population (**Table 1**). To validate these candidate SplB epitopes, we expanded peripheral blood T cells in the presence of SplB for three weeks and assessed their proliferative capacity after restimulation with either single peptides or the whole SplB protein. Peptides SplB_4–18_ and SplB_10–24_ were not included in these assays, as their strong hydrophobic nature inhibited synthetization.

**Table 1:** Epitope prediction of immunodominant 15-mer SplB peptides.

Name	Sequence	Epitope prediction		
		ø Percentile_rank (IEDB)	ø Score (SYFPEITHI)	HLA-type
SplB_1–15_	MNKNVVIKSLAALTI	1.3	17.5	HLA-DRB1*01:01; *04:01;*11:01;*13:01; *15:01
SplB_4–18_	NVVIKSLAALTILTS	1.4	22.3	HLA-DRB1*01:01; *04:01;*11:01;*13:01; *15:01
SplB_7–21_	IKSLAALTILTSVTG	3.6	20.5	HLA-DRB1*01:01; *04:01;*11:01;*13:01; *15:01
SplB_10–24_	LAALTILTSVTGIGT	5.0	22.5	HLA-DRB1*01:01; *04:01;*11:01;*13:01; *15:01
SplB_51–65_	YTGVVAFKSATGFVV	2.7	18.8	HLA-DRB1*01:01; *04:01;*11:01;*13:01; *15:01
SplB_54–68_	VVAFKSATGFVVGKN	3.9	20.5	HLA-DRB1*01:01; *04:01;*11:01;*13:01; *15:01
SplB_60–74_	ATGFVVGKNTILTNK	6.2	17.5	HLA-DRB1*01:01; *04:01;*11:01;*13:01; *15:01
SplB_96–110_	NGGIYSIKKIINYPG	3.4	20.5	HLA-DRB1*15:01;*11:01
SplB_99–113_	IYSIKKIINYPGKED	5.3	18.7	HLA-DRB1*04:01; *11:01;*15:01
SplB_137–151_	VTPFKYAAGAKAGER	4.1	22.3	HLA-DRB1*01:01; *04:01;*11:01
SplB_162–176_	KNKYVLYESTGPVMS	4.5	16.0	HLA-DRB1*04:01;*11:01
SplB_179–193_	GSSIVYSAHTESGNS	5.8	20.0	HLA-DRB1*04:01; *11:01;*13:01
SplB_219–233_	RNAYGVYFTPEIKKF	7.1	14.5	HLA-DRB1*11:01;*15:01

**Figure 4:**
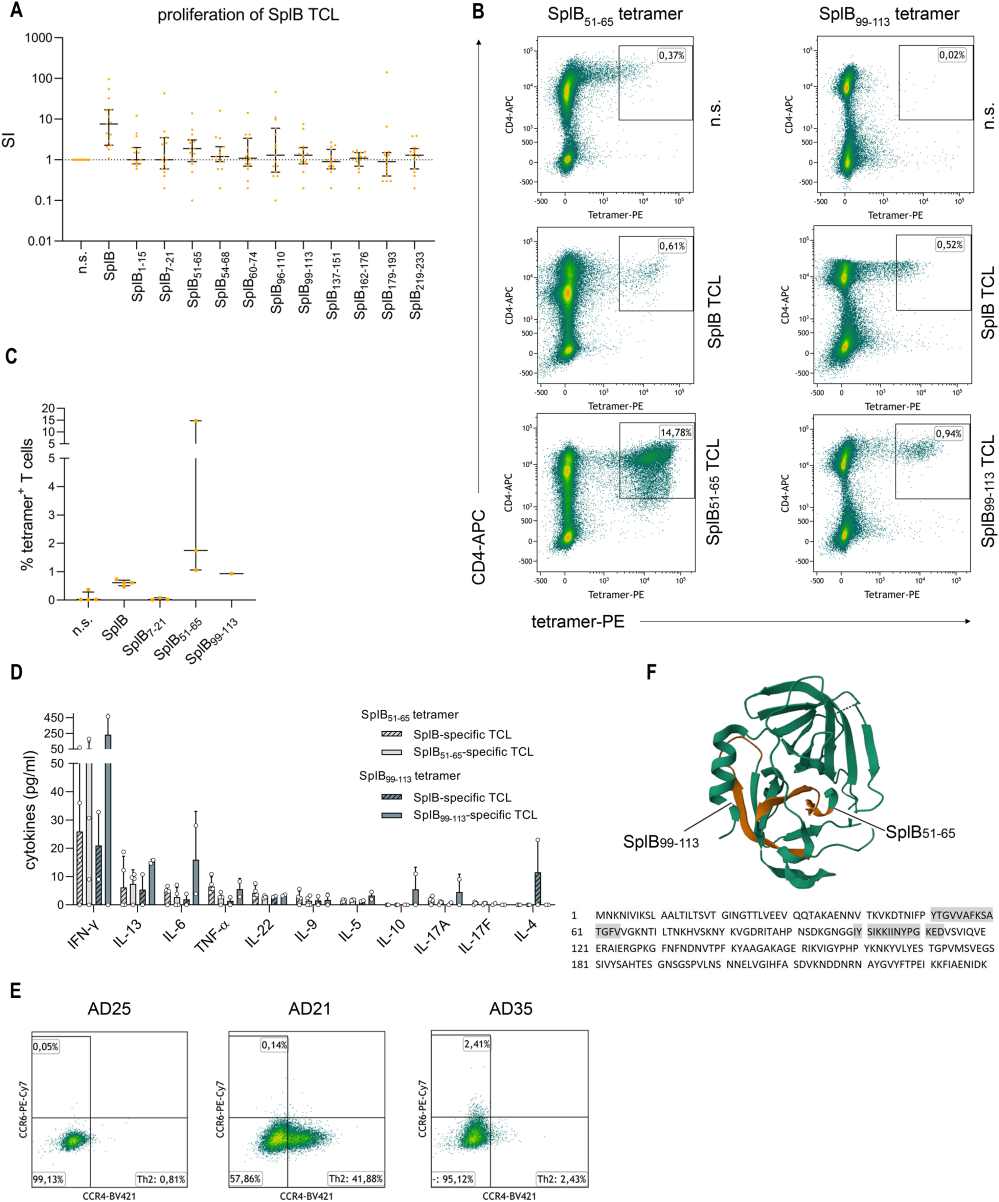
Prediction and validation of SplB epitopes. **(A)** *In silico* predicted epitopes were synthesized and tested for proliferation by ^3^H-thymidine incorporation on SplB-specific T cell lines of AD patients (n = 15), which were generated by stimulations of PBMCs with SplB for three weeks for specific T cell expansion. Stimulation index (SI) is calculated as the ratio of protein or peptide-stimulated to unstimulated control cells. Median and interquartile range are shown. Friedman test. **(B)** MHC tetramer staining of SplB-specific T cell line, exemplarily shown for one SplB-reactive AD patient with HLA-DRB1*15:01 (left) and one SplB-reactive AD patient with HLA-DRB1*11:01 (right). **(C)** Frequency of MHC tetramer^+^ T cells in unstimulated T cells as well as SplB-, SplB_7–21_-, SplB_51–65_-, and SplB_99–113_-peptide T cell lines. **(D)** Cytokine expression capability of sorted MHC tetramer^+^ T cells, after stimulation with concanavalin A for 48 h. MHC-II SplB_51–65_ tetramer n = 4; MHC-II SplB_99–13_ tetramer n = 2 AD patients. Cell-free supernatants were collected and cytokines were analyzes with bead-based cytometry assay. **(E)** Surface expression of CCR4 and CCR6 on SplB-specific MHC tetramer^+^ T cells. **(F)** Predicted molecular structure of SplB with epitopes SplB_51–65_ (YTGCVAFKSATGFVV) and SplB_99–113_ (IYSIKKIINYPGKED) shown in orange. Median with interquartile range are depicted.

Thirteen of 14 patients (93%) showed reactivity against SplB (**Figure 4A**). Most patients tested reacted to the 15-mer peptides SplB_1–15_ (MNKNVVIKSLAALTI), SplB_7–21_ (IKSLAALTILTSVTG), SplB_51–65_ (YTGVVAFKSATGFVV), SplB_96–110_ (NGGIYSIKKIINYPG), SplB_99–113_ (IYSIKKIINYPGKED), SplB_179–193_ (GSSIVYSAHTESGNS), and SplB_219–233_ (RNAYGVYFTPEIKKF). These peptides were subsequently evaluated for their folding into MHC molecules (**Supplementary Table 2**). We focused on HLA-DRB1*11:01 and HLA-DRB1*15:01, as these were the most common HLA types in the cohort. Complexes with good and intermediate folding (SplB_7-21_-HLA-DRB*15:01, SplB_51–65_-HLA-DRB1*15:01, and SplB_99–113_-HLA-DRB1*11:01) were used in further experiments (**Table 1**).

PBMCs were isolated from five patients with AD with matching HLA types and stimulated either with SplB, SplB peptides, or left unstimulated. After 14 days of culture, the cells were stained with the corresponding tetramers (SplB_7–21_-HLA-DRB*15:01, SplB_51–65_-HLA-DRB1*15:01, and SplB_99–113_-HLA-DRB1*11:01). As negative controls, MHC tetramers not matching the donors’ HLA types were used (**Figure 4B**; **Supplementary Figure 4**). T cells from patient AD21 did not proliferate after restimulation, and this patient was excluded from further analysis. These exemplary MHC multimer stainings confirm the immunodominant character observed in **Figure 4A** for the epitopes SplB_51–65_ and SplB_99–113_, but not SplB_7–21_ (**Figure 4C**).

### 4.5 SplB-specific, MHC tetramer^+^ cells are dominated by Th1 and Th2 cells

To characterize the phenotype and cytokine secretion capacity of SplB_51–65_ and SplB_99–113_ specific CD4^+^ T cells, tetramer^+^ T cells were sorted and stimulated with concanavalin A for 48 h. Cytokine secretion was assessed by a bead-based multiplex assay. IFN-γ and IL-13 were the most commonly secreted cytokines (**Figure 4D**), thereby mimicking the response observed in PBMC stimulation (**Figure 3**; **Supplementary Figure 3**). The majority of MHC tetramer^+^ cells were negative for both CCR4 and CCR6, with the exception of one patient who displayed a substantial population of CCR4^+^/CCR6^−^ Th2 cells (**Figure 4E**).

AlphaFold prediction revealed that SplB_51–65_ and SplB_99–113_ are located within β-strands of the protease’s secondary structure (**Figure 4F**) and share sequence similarities with SplA–F, while SplB_51–65_ appears to be more conserved among the Spls than SplB_99–113_ (**Supplementary Figure 5**). Interestingly, SplB shows high homology with the extracellular serine protease V8 protease of *S. aureus* (sspA), and the identified epitopes share three (Y, V, and G in SplB_51–65_) and four amino acids (I, Y, G, and D in SplB_99–113_) in the respective regions (36).

### 4.6 Limited role of CD8^+^ T cells in response to SplB

We further predicted HLA-A*02:01–binding 9-mer epitopes within SplB and synthesized those with the highest scores for experimental validation (**Supplementary Table 3**). Expanded SplB-specific T cell lines showed moderate reactivity to these peptides (**Supplementary Figure 6A**), and some of these peptides showed strong binding to empty HLA-A*02:01 monomers (**Supplementary Figure 6B**). These monomers were tetramerized and used to stain *in vitro* expanded T cell lines (**Supplementary Figure 5C**), but none of the patients’ samples showed substantial numbers of MHC-I tetramer^+^ cells (**Supplementary Figure 5D**). This was corroborated by analysis of the activation marker CD137 on CD8^+^ T cells after stimulation of PBMCs with SplB, which revealed that CD8^+^ T cells are not primarily involved in the adaptive T cell response to SplB in AD patients (**Supplementary Figures 6E, F**). Since we observed cell proliferation after restimulation with these 9-mer peptides, these could represent MHC class II epitopes. However, this observation requires further investigation.

### 4.7 SplB-specific T cells are clonally propagated in the lesional skin of AD patients

**Figure 5:**
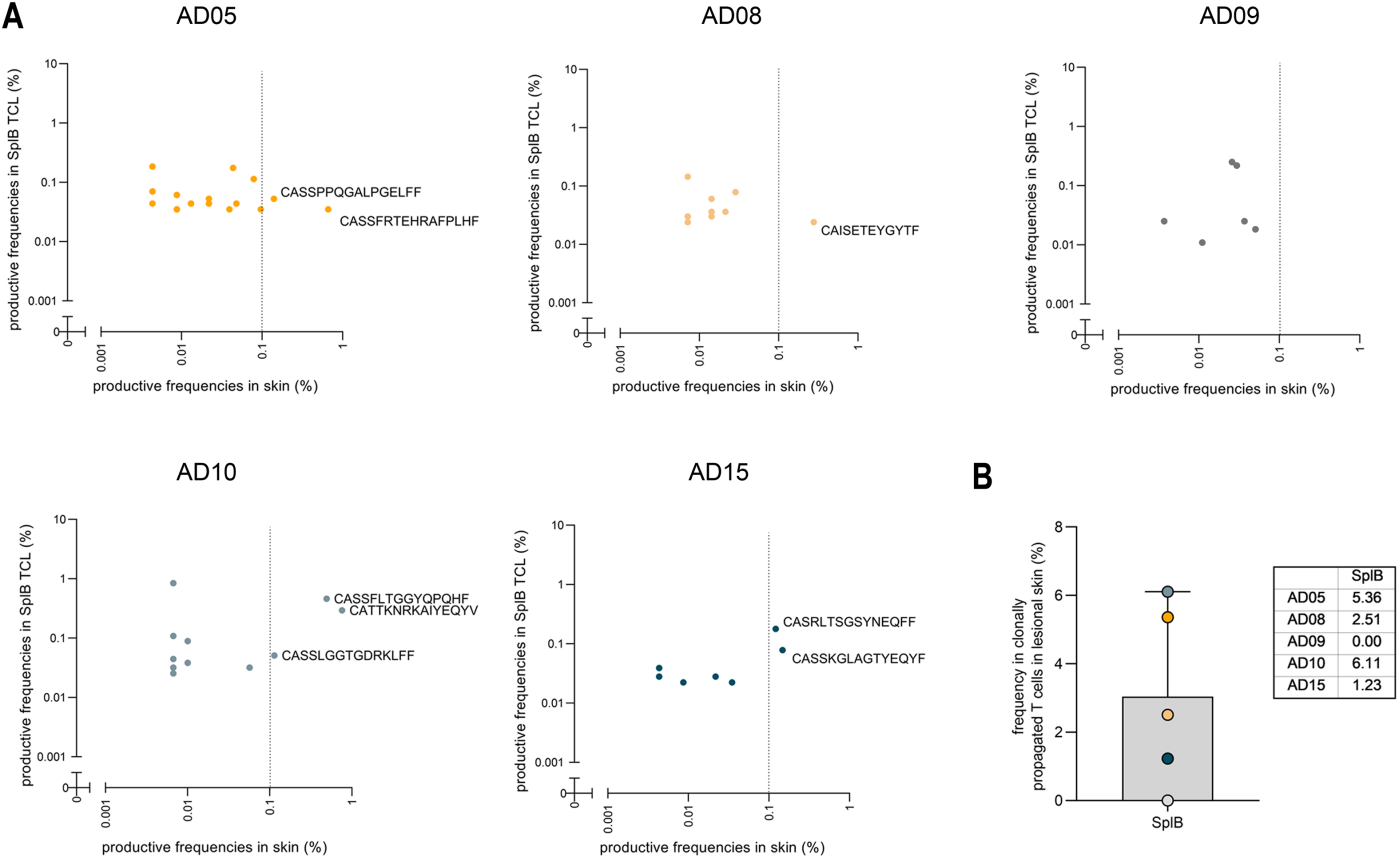
Clonally expanded T cells with specificity to SplB are present in lesional AD skin. To investigate the presence of SplB-specific in the lesional skin of AD patients, T-cell clones were defined as SplB-specific on the basis of SplB-reactive T-cell lines generated from each patient’s blood as described before. If a T-cell clone, defined by its CDR3, was present and propagated in the SplB-reactive T cell line with at least a threefold higher frequency compared to not-stimulated autologous T cell line (>3 clones), or even absent in the latter, it was regarded as SplB-specific. A threshold was defined to focus on clonally-propagated T cells in the skin. T cell clones in the lesional skin with a frequency of >0.1% are referred to as clonally propagated and considered as disease-driving (threshold shown as dashed line). **(A)** Pairwise-scatter blots showing the productive frequencies and sharing of T cell clones in the SplB-specific T cell line and in the lesional skin of the same AD patient of five AD patients (AD05, AD08, AD09, AD10, and AD15). **(B)** Frequency of SplB-specific T cells within the clonally propagated T cell fraction of the skin infiltrate in lesional AD skin. Each data point reflects a single patient.

To investigate the presence of SplB-specific T cells in the lesional skin of AD patients, we searched for overlapping TCRB CDR3 sequences between the SplB-specific T cell lines from peripheral blood and autologous lesional skin biopsies (clinical severity, demographic data, and IgE sensitizations are listed in **Supplementary Table 4**). SplB-reactive T cell lines were generated from each patient’s PBMCs and subjected to sequencing of the TCRB CDR3 region. A T cell clone was considered SplB-specific if it was present in more than three copies in the SplB-reactive T cell line and if its copy number was at least threefold higher than in the unstimulated PBMC culture of the same patient (44). All five patients investigated had detectable SplB-specific T cell clones in lesional skin that shared TCRB CDR3 sequences with those expanded with SplB from peripheral blood (**Figure 5A**; **Supplementary Table 5**). To identify putatively disease-driving, antigen-specific T cells, we next focused on clonally expanded skin T cells: T cells were considered clonally propagated if detectable at a frequency of 0.1% or higher among all clonally expanded T cells in lesional skin. In the five patients tested, SplB-specific T cells represented up to 3.0% ( ± 2.6%) of all clonally expanded T cells in the skin infiltrate (**Figure 5B**).

## 5 Discussion

T cells play a pivotal role in the pathogenesis of AD and respond to microbial antigens, such as proteins of *S. aureus* (45–47). Elucidating the specificity of these T cell responses is critical to identify trigger factors and establish therapeutic targets (46, 47). *S. aureus* antigens have been discussed as putative triggering factors because, although the immune response to microbial antigens is generally type 1/type 3 dominated (48, 49), patients with AD or severe asthma may have elevated levels of IgE or Th2 cell proliferation directed against *S. aureus* factors (23, 50–56). Recently, a murine model of AD was established by applying *S. aureus* strains derived from patients with AD to the ears without further adjuvants, wounding, or stressing. The mice developed skin inflammation driven by myeloid and T cell immune responses, including type-2 cytokines, which led to local tissue memory formation. This model highlights the potential of *S. aureus* to induce type-2 skin inflammation and eczematous lesions (57).

In this study, the cytokine response and phenotype of SplB-specific T cells were not markedly different in AD compared to healthy individuals. In both groups the cytokine response was type 1/type 2 dominated; however, specific serum IgE against Spls—especially SplB—was increased in patients with AD and was detectable only in a few healthy individuals, indicating systemic sensitization in atopic individuals. The family of Spl proteins has been shown to harbor the intrinsic capacity to induce type-2 responses even in the absence of an atopic background, which might represent an evasion strategy to avoid a pronounced antimicrobial type-1/type-3 response (27, 58). *In vivo*, this Th2 reaction may be balanced by a dominant Th1/Th17 antimicrobial response in healthy individuals (49), whereas in patients with AD it occurs in the fertile ground of chronic type-2 inflammation (5). Furthermore, the factors of location and quantity constitute decisive differences when comparing healthy individuals and patients: locally on the lesional AD skin, where type 2 inflammation occurs, the presence of *S. aureus* and thus its proteases is also increased, while *S. aureus* colonizes the skin of healthy individuals only in exceptional cases.

As mentioned earlier, SplB is thought to have an intrinsic type 2 polarizing potential, which may represent a protease-mediated immune escape mechanism to avoid the host’s Th17 immune response. In addition, the induction of type 1 cytokines (IFN-γ, TNF-α, CXCL9, and CXCL10) has also been reported, indicating that SplB can elicit mixed immune responses (58).

Other members of the Spl protease family are associated with type-2 immune responses. For example, SplD and the highly homologous SplF (94.6% sequence similarity) can induce IL-33-driven allergic inflammation and IgE production in a mouse model (25, 28, 30, 59). SplA promotes bacterial invasion by cleaving the epithelial cell surface protein mucin-16 (29, 59, 60).

In our hands, a subset of SplB-specific CD154^+^CD4^+^ T cells expressed CCR6, which has been described as a Th17 polarization surface marker, and low numbers of IL-17A-producing cells were detected by ELISpot in response to SplB. Although this likely reflects a physiological response to bacterial antigens, Th17 cells have been described in lesional skin in patients with AD, especially in children and certain endotypes (61, 62). However, their pathogenic relevance remains controversial, as clinical trials targeting IL-17 have not shown clear efficacy; however, they might have therapeutic potential in certain AD patient subgroups (10, 63).

Immune responses to bacterial proteases are not restricted to *S. aureus*; proteases from *S. epidermidis* have also been shown to induce type 2 immune responses in AD and disrupt epithelial barriers and induce skin inflammation as shown in a mouse model (64–68). These observations support the broader concept that microbial proteases can act as drivers of allergic inflammation in barrier tissues. Future studies could address whether Spl proteases induce epithelial-derived cytokines, such as IL-33 or thymic stromal lymphopoietin (TSLP), or pruritogenic cytokines, including IL-31, and whether SplB directly affects skin barrier integrity, as proteases are known to play a role in the penetration of the epidermis (69, 70).

In this work, we discovered two T cell epitopes in SplB for HLA-DRB1*11:01 and *15:01. Owing to the heterogeneity of the patients’ HLA backgrounds, the experiments involving MHC tetramer staining of peripheral blood mononuclear cells (PBMCs) and skin were merely a proof of concept. To identify T cell epitopes as *S. aureus* vaccine candidates, an earlier study combined peptide epitope prediction by the NetMHCII server (71) with molecular docking of proteins contained in the *S. aureus* secretome (72, 73). Thus, the authors identified T cell epitopes in different *S. aureus* proteins, including YNNGGFYKV from SplE. The short sequence FYKV overlaps with SplB_60–74_, suggesting that this region in the primary structure is immunodominant. Generated SplB_7–21_ MHC tetramers did not bind CD4^+^ T cells of AD patients, which is likely because this peptide includes the N-terminal signal peptide of the protein that is cleaved after Ala_36_ during the secretion process by a signal peptidase (26, 74).

Furthermore, for the first time, we analyzed the prevalence of SplB-specific CD4^+^ T cells in lesional AD skin and detected such T cells in four out of five AD patients. This dual TCR sequencing approach has been used earlier in a proof-of-concept study to investigate aeroallergen-specific T cells in the lesional skin of AD patients. Up to 28% of clonally propagated T cells were annotated as specific for aeroallergens, namely house dust mite, grass pollen, birch pollen, and rye pollen (44). The presence of expanded SplB-specific T cells in lesional skin suggests that this antigen may contribute to local inflammation in AD. Future studies might confirm the specificity of T cells, for example, by expressing the alpha and beta chains of the identified TCR in reporter cells (75). The impact on the disease severity may be further investigated by stimulating mice that are sensitized to *S. aureus* and display skin lesions (compare (57)) with Spl proteins.

In conclusion, we described a pronounced type 1/type 2 response to SplB, resembling the phenotype described for chronic lesions, and elevated numbers of SplB-specific T cells in lesional skin. These observations indicate a potential role for *S. aureus* SplB in the pathogenesis and chronification of cutaneous type-2 inflammation, emphasizing the crucial role of the microbiome in AD pathogenesis. This finding also provides a rationale for targeting the microbiome in addition to the type 2 response in AD therapy.

## Data Availability

The datasets presented in this study can be found in the online repository European Genome-Phenome Archive, https://ega-archive.org, under the study number 3344 and the analysis number 84410.
