# Supplementary material for: Staphylococcus aureus serine protease-like protein B elicits a type 1/type 2 immune response in atopic dermatitis patients
Source: Front Immunol. 2026 Jun 4;17:1798583. doi: 10.3389/fimmu.2026.1798583 (PMC13275367; doi:10.3389/fimmu.2026.1798583)
Supplement: Supplementary file 2 [file SupplementaryFile1.docx]

Supplementary Material

# Supplementary Methods

## Isolation and culture of peripheral blood mononuclear cells

Peripheral blood mononuclear cells (PBMCs) were isolated by Ficoll density centrifugation (PAN-Biotech GmbH, Aidenbach, Germany). 1x10^6^ PBMCs per ml were seeded into 24 well plates in Iscove’s medium (Biochrom KG, Berlin, Germany) supplemented with 4 % heat-inactivated human AB serum (PAN-Biotech GmbH, Aidenbach, Germany), non-essential amino acids, 100 µg/ml penicillin/streptomycin, 50 µg/ml gentamycin and 2 mM L-glutamine (Bio&SELL GmbH, Feucht bei Nürnberg, Germany) (termed Iscoves/AB, IAB) and incubated in a humidified cell incubator at 37 °C and 5 % CO_2_.

## Proliferation testing

Generated T cell lines were collected, washed and transferred with a density of 5x10^3^ cells / 50 µl IAB in triplicates into a 96-well plate. 7.5x10^4^ autologous irradiated (55 Gy) PBMCs in 100 µl and SplB (2.5 µg/ml) or SplB peptides (10 mg/ml) in 50 µl IAB were added, respectively. After 5-7 days, ^3^H-thymidine (18.5 kBq/well) was added and incubated overnight at 37 °C. Cells were harvested and ^3^H-thymidine uptake was measured with a beta counter (Microbeta2, Perkin Elmer, Waltham, MA, USA). Cells with a proliferation fold-change of ≥1.5 after stimulation with SplB were considered a SplB-specific T cell line.

## Multiplex immunoassay

From AD patients and healthy individuals obtained PBMCs, monocytes were isolated with a CD14+ magnetic bead-based Isolation kit (Miltenyi, Bergisch Gladbach, Germany) and irradiated with 55 Gy. T cells were isolated with a magnetic bead-based Pan T cell isolation kit (Miltenyi, Bergisch Gladbach, Germany). 1x10^5^ T cells with 1x10^4^ APCs were cultivated with recombinant SplB (2.5 µg/ml) or left unstimulated (n.s.) for 9 days. At day 5, 10 U/ml rh-IL-2 (Merck, Darmstadt, Germany) was added to the culture. Supernatants were collected and stored immediately at -80 °C. A bead-based immunoassay was used to quantify released T cell cytokines (LEGENDplex™, Biolegend San Diego, CA, USA): The amount of T cell cytokines (IL-2, IL-4, IL-5, IL-6, IL-9, IL-10, IL-13, IL-17A, IL-17F, IL-21, IL-22, TNF-α and IFN-γ) were analysed by the Human Th Panel (13-plex, 740722) according to the manufacturer’s instructions.

## Nucleic acid isolation

Skin samples were homogenized applying the TissueRuptor (Qiagen, Venlo, Netherlands). Genomic DNA of T cell lines and homogenized skin samples were isolated and purified using the NucloeoSpin® Tissue kit (740952.50, Machery-Nagel, Düren, Germany) according to the manufacturer’s instruction.

## Enzyme-linked immunosorbent spot assay

ELISpot for IL-17A was performed according to manufacturer´s instructions (Mabtech AB, Nacka Strand, Sweden). Unspecific binding sites were blocked with Iscove´s medium with 10 % heat-inactivated human AB serum. 2x10^5^ PBMCs and 2.5 µg/ml SplB in 200 µl Iscove´s medium with 4% human AB serum were seeded. Medium served as negative control, 10 µg/ml ConA in 100 µl as positive control. PBMCs were stimulated for 48 h at 37 °C. Spots were quantified with an ELISpot reader (AID Autoimmun Diagnostika GmbH).

## Cell sorting of MHC tetramer positive T cells

Live CD4+ tetramer+ T cells were sorted on a FACSAria™ Fusion in the Hannover Medical School Research Core Facility Cell Sorting. The sorted cells were stimulated with the mitogen concanavalin A (10 µg/ml, ConA, Merck, Darmstadt, Germany) for 48 h at 37 °C. Supernatants were collected and cytokine secretion was investigated with a bead-based immunoassay like mentioned before.

# Supplementary Figures


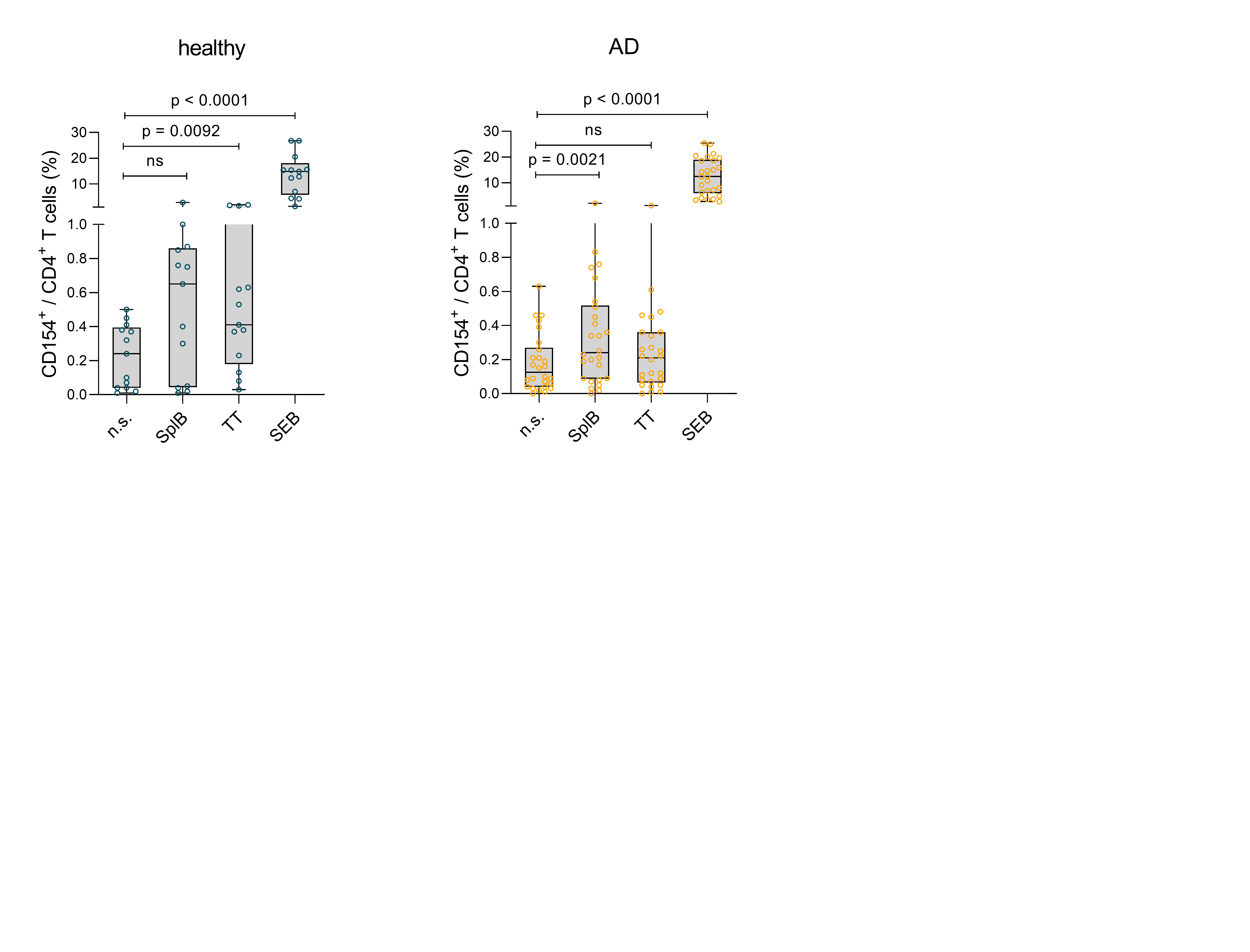


**Supplementary Figure 1. Adaptive T cell response to SplB.**

PBMCs were isolated from healthy donors and AD patients. 1x10^6^ PBMCs were stimulated with SplB in the presence of anti-human CD40 and anti-human CD28 for 24 h. CD154+ frequencies among CD4+ T cells in healthy adults (n=13, blue) and AD patients (n=27, yellow), respectively. Friedman test with Dunn’s multiple comparison test. TT, tetanus toxoid, SEB, *S. aureus* enterotoxin B.

**
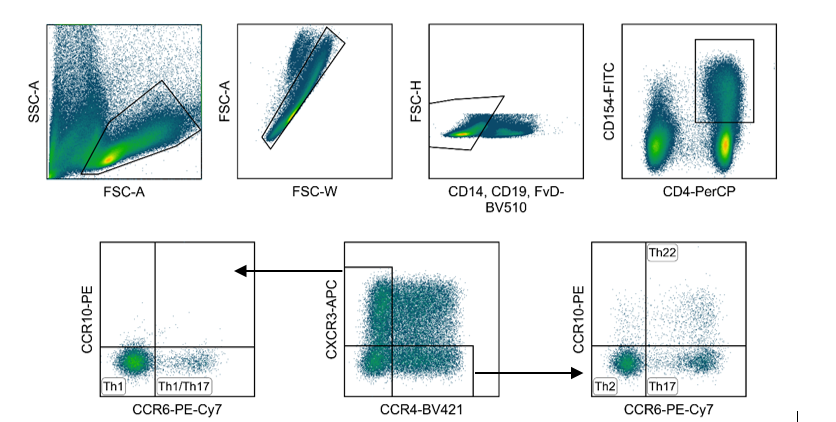
**

Supplementary Figure 2. Gating strategy to characterize SplB-specific CD4+ T cells

PBMCs were isolated from healthy individuals and AD patients. 1x10^6^ PBMCs were stimulated for 24 h in the presence of anti-human CD40 and anti-human CD28 antibodies. Expression of CD154 on CD4+ T cells as well as chemokine receptors (CCR4, CCR6, CCR10, CXCR3) to characterize T cell polarization were investigated with flow cytometry.

**
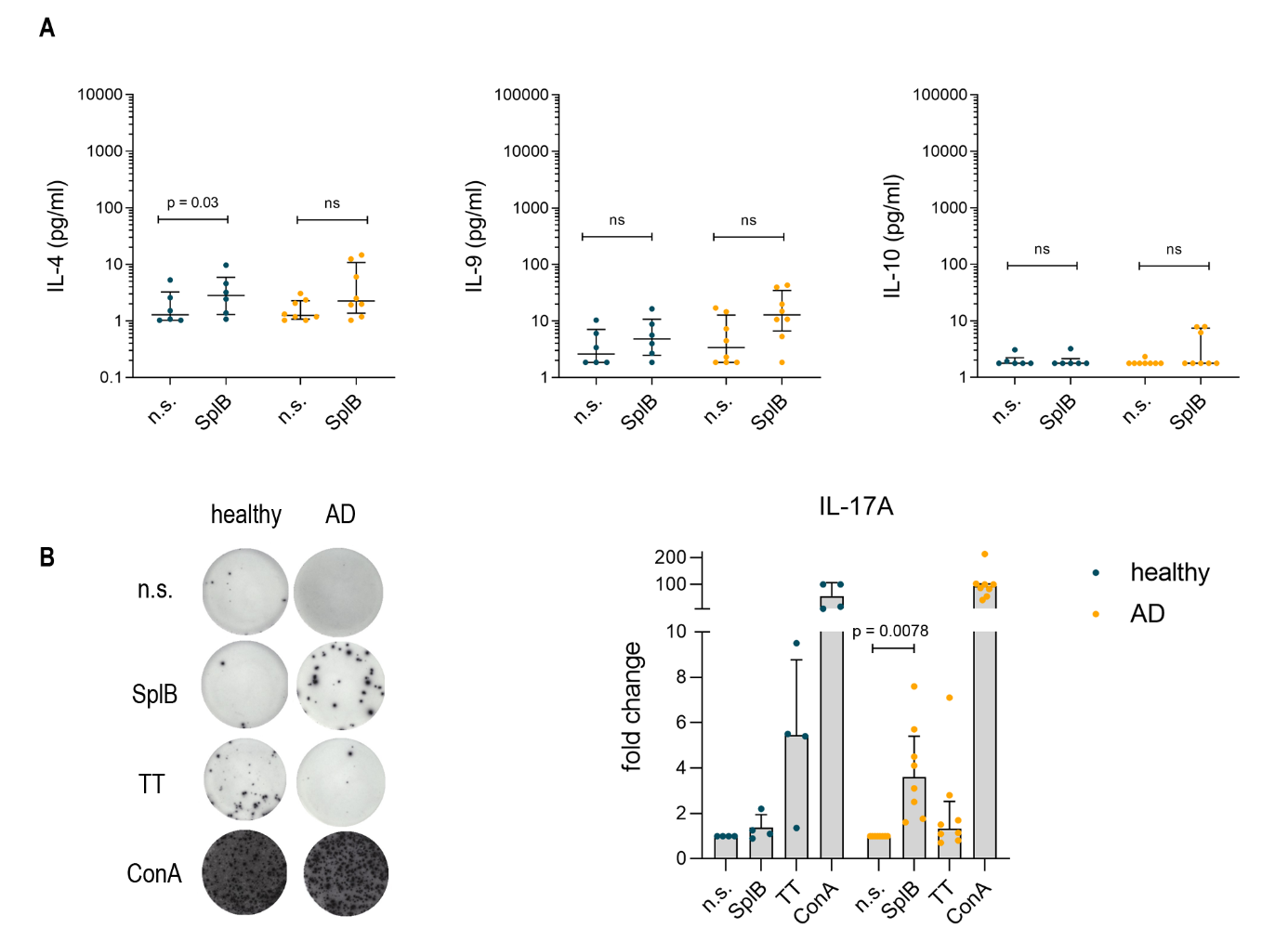
Supplementary Figure 3. Cytokine secretion in response to SplB.**

(A) T cells and monocytes were isolated from PBMCs of AD patients (n=8, yellow) and healthy individuals (n≥5, blue) and stimulated with recombinant SplB. IL-2 was added on day 7 and supernatants were collected on day 9. Cytokines as depicted were measured by bead-based cytometry assay. Wilcoxon test to compare SplB stimulated cells with unstimulated control. Median with interquartile range are depicted. *p < 0.05, **p < 0.01, ***p < 0.001. (B) ELISPOT analysis of IL-17A. 2x10^5^ PBMCs of healthy individuals and AD patients were stimulated with SplB for 48 h and IL-17A–secreting cells were detected by ELISPOT assay using an anti–IL-17A detection antibody. Spots represent individual IL-17A–producing cells. TT, tetanus toxoid, ConA, concanavalin A.

**
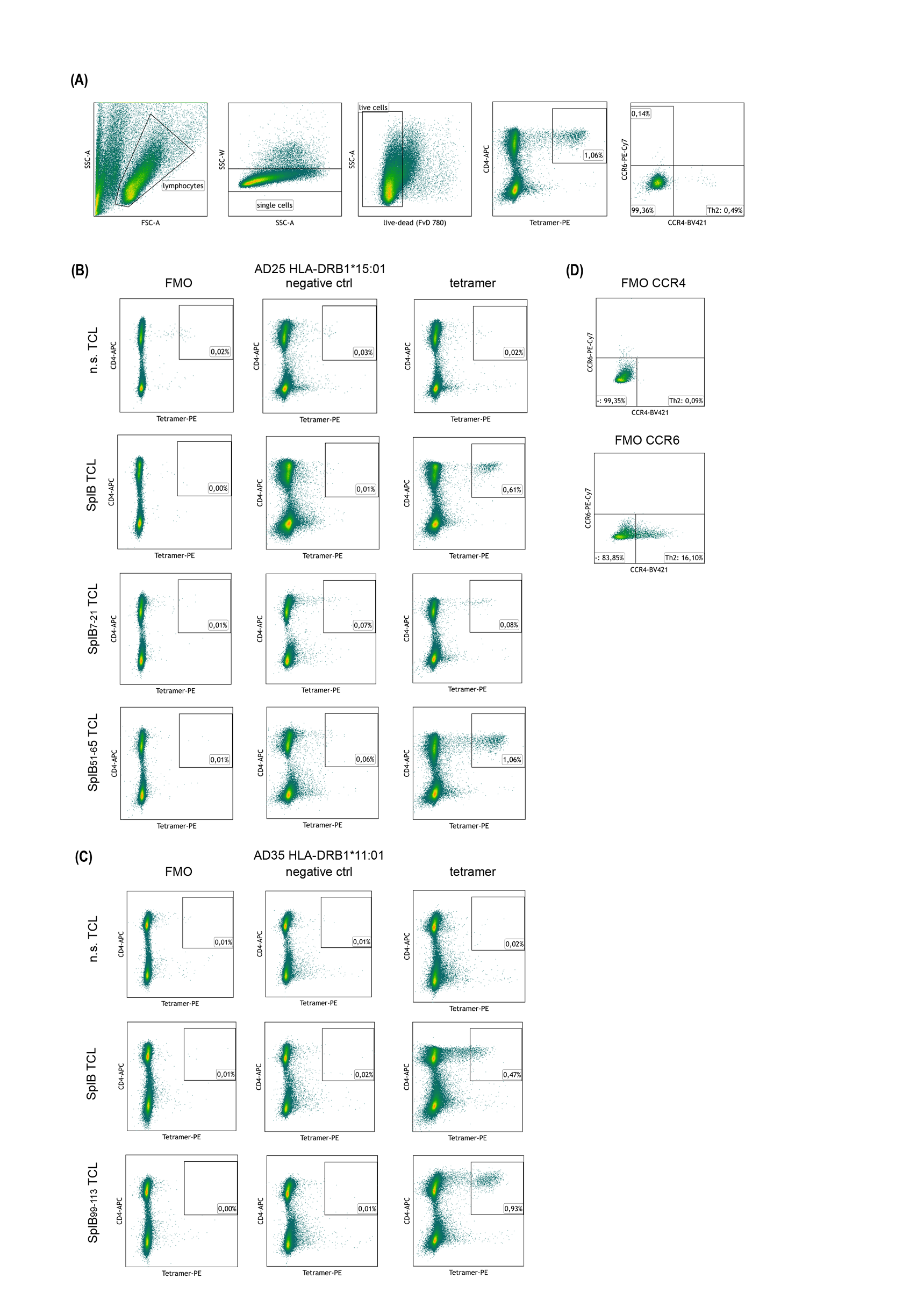
**

**Supplementary Figure 4. Gating and staining of T cell lines (TCL) with MHC class II tetramers.**

(A) Gating strategy for MHC class II tetramer staining of SplB specific TCL. (B) Exemplarily AD patient with HLA-DRB1*15:01. Unstimulated TCL, SplB TCL, SplB_7-21_ TCL and SplB_51-65_ TCL were stained with tetramers for HLA-DRB1*15:01. (C) Exemplarily AD patient with HLA-DRB1*11:01. Unstimulated TCL, SplB TCL and SplB_99-113_ TCL were stained with tetramers for HLA-DRB1*11:01. (D) FMO controls for CCR4 and CCR6 staining. FMO, fluorescence minus one control; negative control, tetramer not matching the HLA-type.

**
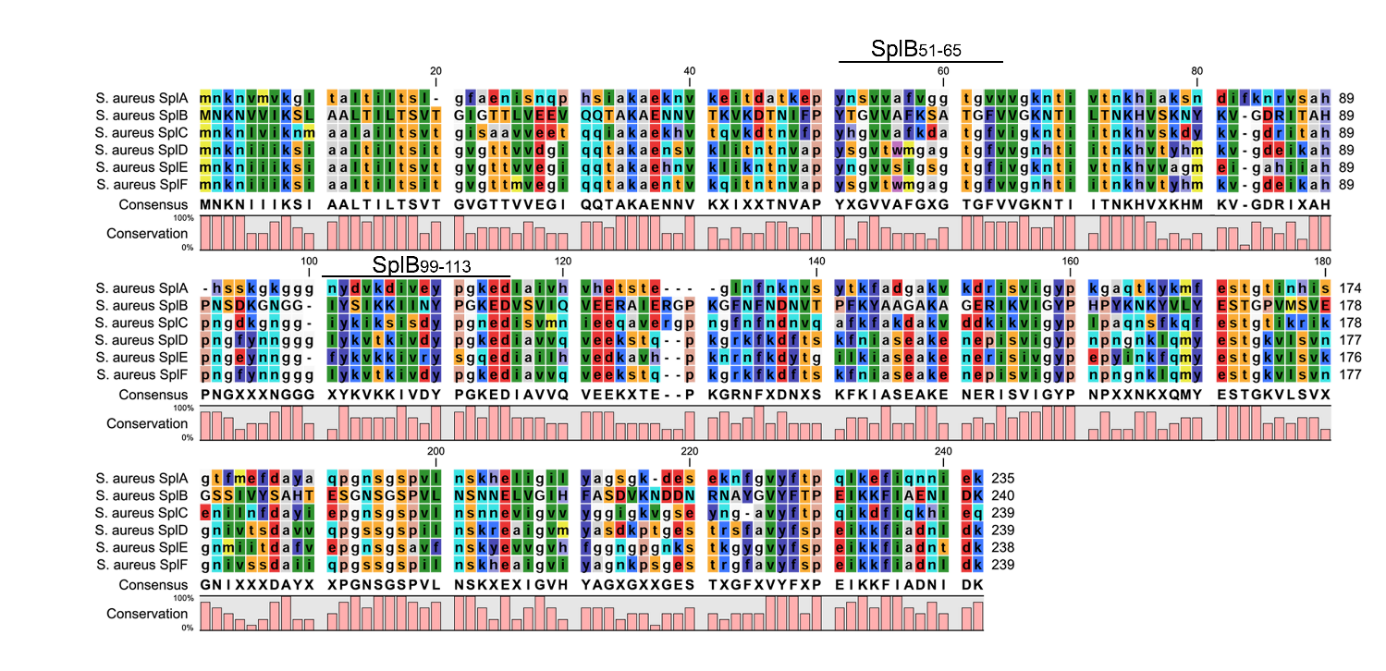
Supplementary Figure 5. Sequence alignment of SplA-F.**

Sequences of SplA-F were aligned and immunodominant epitopes of SplB SplB_51-65_ and SplB_99-113_ are marked. SplB_51-65_ shares up to 13 amino acids with other Spls (SplA 9/15, SplC 13/15, SplD 8/15, SplE 9/15 and SplF 8/15). SplB_99-113_ shares up to 10 amino acid with other Spls (SplA 9/15, SplC 10/15, SplD 9/15, SplE 7/15, SplD 9/15).

**
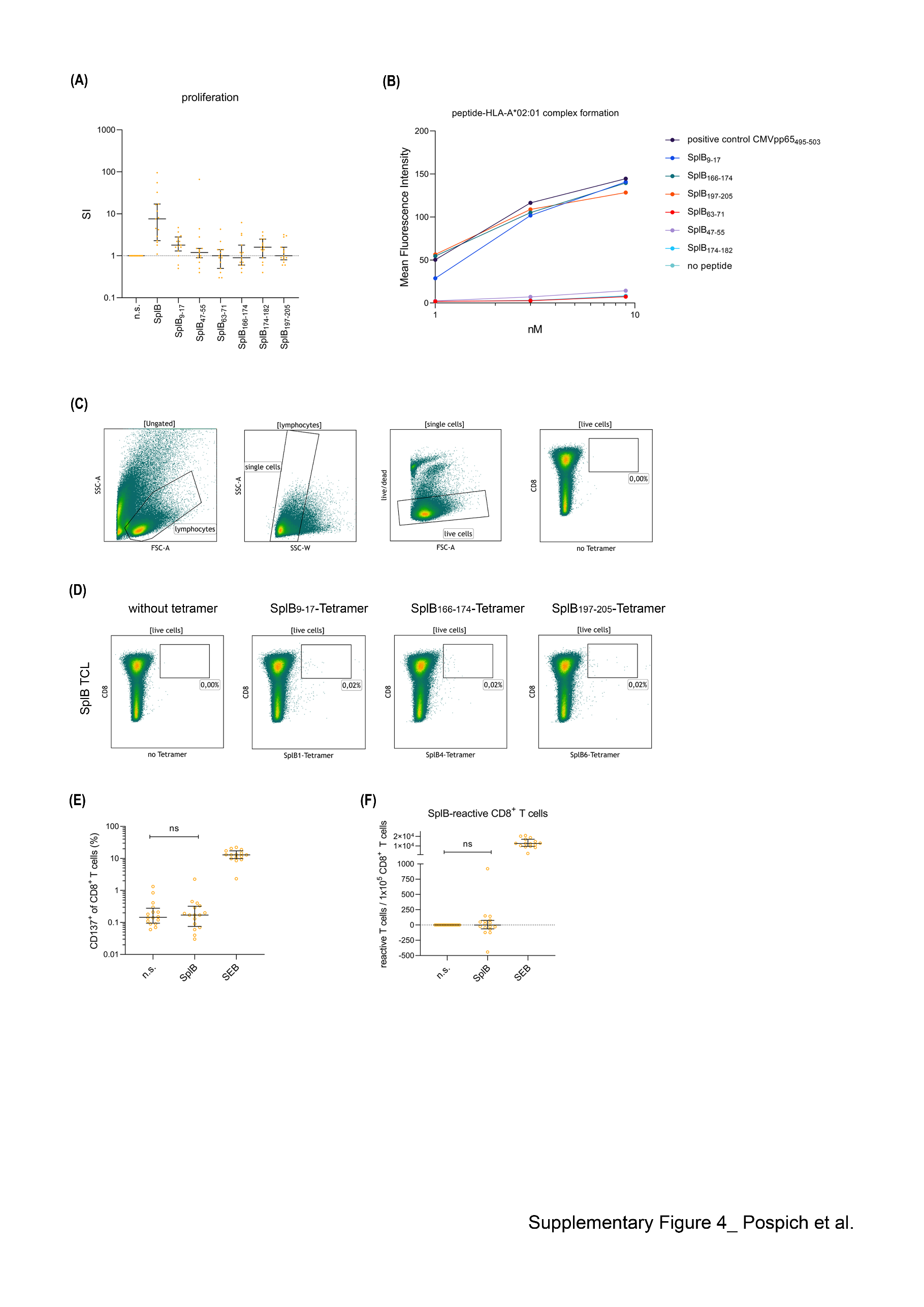
Supplementary Figure 6. Prediction of MHC class I epitopes.**

(A) *In silico* predicted epitopes were synthesized and tested for proliferation by ^3^H- thymidine intake on SplB-specific TCLs of AD patients (n=15). Stimulation index (SI) is calculated as the ratio of protein or peptide-stimulated and unstimulated control cells. Median and interquartile range are shown. (B) Peptide-HLA-A*02:01 complex formation was investigated. CMVpp65_495-503_ was used as positive control. SplB_9-17_, SplB_166-174_ and SplB_197-205_ bind to HLA-A*02:01, while SplB_63-71_, SplB_47-55_ and SplB_174-182_ did not bind. For SplB_9-17_, SplB_166-174_ and SplB_197-205_ HLA-A*02:01 tetramer were synthesized. (C) Gating strategy for MHC class I tetramer staining of SplB specific TCL. (D) Staining of SplB TCL with HLA-A*02:01 tetramers. No tetramer positive cells were found. (E) Investigation of activation of CD8+ T cells. 1x10^6^ PBMCs were isolated from AD patients (n=16) and stimulated with 2.5 µg/ml SplB. The expression of CD137 on CD8+ T cells were investigated. Friedman test with Dunn’s multiple comparison test revealed no differences between unstimulated T cells and SplB stimulated T cells. (F) SplB reactive CD8+ T cells. Friedman test with Dunn’s multiple comparison test revealed no differences. Median with interquartile range are depicted.
